# Effects of Coping Strategies on Health-Related Quality of Life of People with Neuromuscular Diseases

**DOI:** 10.3390/muscles3020011

**Published:** 2024-04-03

**Authors:** Irune García, Alicia Aurora Rodríguez, Corrado Angelini, Maddalen García-Sanchoyerto, Patricia Espinosa-Blanco, Oscar Martínez

**Affiliations:** 1 Neuro-e-Motion Research Team, Faculty of Health Sciences, University of Deusto, Avda. Universidades 24, 48007 Bilbao, Spain; aliciarodriguez.b@deusto.es (A.A.R.); garcia.maddalen@deusto.es (M.G.-S.); patricia.espinosa@deusto.es (P.E.-B.); oscar.martinez@deusto.es (O.M.); 2 Neuromuscular Laboratory, Department of Neurosciences, University of Padova, Campus Biomedico Pietro d’Abano, 35131 Padua, Italy; corrado.angelini@unipd.it

**Keywords:** neuromuscular diseases, rare diseases, coping strategies, health-related quality of life, disability, clinical practice

## Abstract

Neuromuscular diseases (NMD) cover a broad spectrum of different rare diagnoses in which the primary lesion is in the peripheral nervous system. The impairment caused by an NMD does not only interfere with physical status but also has a clear impact on health-related quality of life (HRQoL). It is therefore essential to know the coping style used by these patients. This study aims to analyze the coping strategies in a sample of people with NMD and how their coping style affects their HRQoL. This cross-sectional study included 61 adult patients diagnosed with a rare NMD. WHO-DAS II, SIP, SF-36, and COPE-60 instruments were administered. The results showed that people affected by NMDs tend to use more frequent coping strategies such as active planning, personal growth, and acceptance. In contrast, the least-used strategies were restraint, mental disengagement, venting, humor, and religion, which affected HRQoL negatively. Moreover, the degree of disability was a relevant variable, with an impact on HRQoL. Social support can be considered the main coping strategy that leads to an improvement in the psychosocial HRQoL (β = 503, *p* < 0.001). These findings are relevant to clinical practice, given the need to understand the coping variable to improve HRQoL.

## 1. Introduction

Neuromuscular diseases (NMDs) cover a broad spectrum of different rare diagnoses and pathologies in which the primary lesion is in the peripheral nervous system, specifically, in the anterior horn cell, peripheral nerve, neuromuscular junction, or in the muscle [1,2,3]. Being a heterogeneous group of conditions, the idiosyncrasy of each one can be observed in the different etiology, inheritance, incidence, prevalence, classification, age of onset, and prognosis of each person [4,5]. 

However, the main signs of all NMDs consist of progressive muscle weakness and abnormal function. Moreover, NMDs are characterized by their chronicity, low prevalence, progressive disability, a life accompanied by significant comorbidities (e.g., fatigue, respiratory and cardiac complications, muscular pain, etc.), and the absence of a definitive cure [1,5]. 

The impairment caused by an NMD also interferes with the physical status of the patients. The need for assistance from others, the functional disability for performing daily activities, and the delimitation of social participation also have clear impacts on a person’s health-related quality of life (HRQoL) [2,6,7,8,9,10]. HRQoL is defined as “how well a person functions in their life and his or her perceived well-being in physical, mental, and social domains of health” [11]. In this regard, as living with a chronic condition could produce a detrimental effect on HRQoL [12], it is essential to assess the role played by the coping style adopted by these patients in each situation based on their resources and capacities [13,14,15,16,17,18,19,20]. 

According to Lazarus and Folkman’s transactional theory, the coping style refers to both cognitive and behavioral efforts exerted by an individual to respond to and manage specific external and/or internal demands that are perceived as being superior to the personal resources available to them [21,22]. Accordingly, two groups of coping strategies are considered: problem-centered coping strategies and emotion-centered coping strategies [21,22,23]. Problem-centered coping strategies seek to change or manage the problem that is generating distress by modifying it or providing new resources for it (e.g., finding alternative solutions), while emotion-centered coping strategies seek to regulate or alleviate the emotion generated by the problem (e.g., seeking social support, acceptance, denial, support in religion, avoidance, etc.) [21,22].

The relevance of the relationship between HRQoL and coping styles in patients with muscular dystrophies has been suggested [24,25], but there is still a lack of related scientific literature in the broad group of NMDs. Moreover, only very few studies have devoted attention to these two aspects and coping strategies in particular. 

A study conducted on patients affected by myotonic dystrophy type 1 (DM1) or Steinert’s dystrophy revealed that emotion-centered strategies are more prevalent than problem-centered ones [9]. Similarly, some studies [26,27] carried out on amyotrophic lateral sclerosis (ALS) patients showed that these patients used various strategies indistinctly. In particular, acceptance, active coping, planning, positive reinterpretation, and growth were the most commonly used and had an important effect on increasing survival in ALS patients. However, a previous study by Hecht et al. [28] also conducted on adult ALS patients revealed that rumination and seeking support in religion were the primary coping strategies for them. Finally, in a case of a study of patients with myasthenia gravis (MG) [29], the authors reported that an optimistic coping style was the most widely used and was effective in these cases. Likewise, using humor and maintaining positive thinking were prevalent strategies. 

Although the above studies show variability in terms of existing knowledge on this matter, three additional assumptions demonstrate the need to assess coping strategies in the population with NMDs. First, there is a clear lack of quality studies evaluating HRQoL and coping strategies in patients affected by different NMDs [30,31,32]. The studies conducted in this field have been performed with specific conditions [24,25] and, therefore, are not integrated by a wide range of diagnoses that represent the heterogeneity of the NMD group. Moreover, the available studies focus on coping strategies or quality of life descriptively, without exploring in detail the relationships that may exist. Finally, it is up to clinicians to know the coping style used by these patients in their specific situations to be able to assist them in their psychosocial adjustment and improve their coping capacity [27]. Thus, the novelty in this research is to examine the coping style, considering different strategies, and to determine how this affects HRQoL in people with different diagnoses of NMD.

Therefore, this study aimed to analyze the coping strategies used in a sample of people with NMD. Likewise, the influence of different coping strategies and degrees of disability on their HRQoL were also examined. 

## 2. Methods

### 2.1. Participants

A total of 61 Spanish patients (38 women and 23 men) with a low prevalence NMD were included in this study. All the participants were aged between 19 and 77 years and were recruited through various Spanish patient associations: Asociación Miastenia de España (AMES), Bizkaiko Eritasun Neuromuskularren Elkartea (BENE), Federación Española de Enfermedades Neuromusculares (ASEM), and also, from the Hospital Universitario de Cruces, and the Hospital de Basurto.” Table 1 shows the sociodemographic and clinical data. 

The inclusion criteria were: (a) being of legal age; (b) having a diagnosis of a low prevalence of NMD made by a specialist; and (c) having Spanish as one of their main languages. The exclusion criterion was: (a) the presence of any mental psychopathology.

Before its implementation, all the participants completed an informed consent form and agreed to participate voluntarily in the study. The project was approved by the Ethics Committee at the University of Deusto and was conducted following the ethical principles established by the Declaration of Helsinki.

### 2.2. Instruments

The following instruments were used to assess the subjects’ disability, coping strategies, and HRQoL. All of them are validated to the Spanish population and present adequate psychometric properties. Moreover, they have been widely used in the NMD population [33,34,35], and, therefore, their use is justified due to their ability to analyze these variables in the target group.

#### 2.2.1. Disability

The World Health Organization Disability Assessment Questionnaire (WHO-DAS II) ([36]; Spanish Version: [37]) aims to assess disability, considering impairments (discomfort or pain when carrying out an activity, increased effort, having to change how an activity is performed, or having to perform it more slowly) related to various health conditions (illnesses, mental problems, injuries, etc.) experienced over the last 30 days. The 36-item version was used, in which six domains were assessed (ability to move around in their environment, life activities, self-care, understanding and communicating, getting along with people, and participation in society). The responses based on the level of difficulty experienced in each of the activities for each domain are coded using a Likert scale: 1 (none), 2 (mild), 3 (moderate), 4 (severe), and 5 (extreme/unable). The minimum score is 0 and the maximum is 100, with a higher score corresponding to a greater degree of disability. Regarding internal consistency, Cronbach’s alpha values were above 0.70. The International Classification of Functioning, Disability, and Health (ICF) was used to classify the participants into different disability groups.

#### 2.2.2. HRQoL

The Sickness Impact Profile (SIP) ([38]; Spanish Version: [39]) is a test of HRQoL. It provides a measure of the impact produced in daily life by a given disease, since it analyzes the changes in a person’s behavior due to their sickness. The SIP is made up of 136 items through which the person shows their current health condition. The items are divided into 12 categories: sleep and rest, nutrition, intellectual activity, emotional activity, care and body movement, household tasks, work, leisure and hobbies, travel, mobility, social relations, and communication. The minimum score that can be obtained on the test is 0 and the maximum is 100. A higher score means a greater impact and a worse health condition. The Spanish version presents a Cronbach’s alpha of 0.95. 

The 36-item Short-form Health Survey (SF-36) ([40]; Spanish Version: [41]) is a widely used test of HRQoL. This instrument aims to evaluate a person’s health condition and is structured into 36 items that are grouped into 8 health-related dimensions: physical function, body pain, role limitations due to physical problems (physical role), social function, role limitations due to emotional problems (emotional role), vitality, mental health, and general health. The questionnaire scores range from 0 to 100 points, with 0 being the worst health condition and 100 the best. The internal consistency of the Spanish version obtained a Cronbach alpha of 0.70, except in the social function dimension, which was 0.45. 

#### 2.2.3. Coping Strategies

Coping Orientations to Problems Experienced (COPE-60) ([42]; Spanish Version: [43]) is a self-reporting measure that evaluates the different coping strategies used to deal with stress. It has 60 items, which are scored on a Likert scale from 1 (minimum) to 4 (maximum). The COPE-60 comprises 15 subscales, which refer to the different forms of coping: seeking social support, religion, humor, substance use, planning, suppression of competing activities, venting, acceptance, denial, restraint, active coping, personal growth, positive reinterpretation, behavioral disengagement, and mental disengagement. The internal consistency of the test ranges from 0.45 to 0.92 points. 

### 2.3. Procedure

A cross-sectional study was conducted using a convenience sample, considering the low prevalence of the present clinic population. Patient associations disseminated the information, and the patients who were interested in participating were contacted by the researchers. The sessions were individual and conducted by a psychologist. After accepting the informed consent, clinical and socio-demographic data were collected through a brief interview. This was followed by the administration of the different tests described in the previous section. Each session lasted approximately one hour per participant. All the assessments were performed under similar environmental conditions. 

### 2.4. Data Analysis

Statistical analyses were conducted using the statistical software SPSS (Statistical Package for the Social Sciences) version 28.0. The Kolmogorov–Smirnov test was applied before the analyses to determine the normal distribution of the variables. Descriptive statistics were used to describe the participants. Continuous variables were described using means and standard deviations and categorical variables by frequencies and percentages. Spearman’s Rho statistic was determined to analyze the correlation between different coping strategies and HRQoL scores.

Finally, multiple regression analyses were conducted between the degree of disability and different coping strategies and the HRQoL scores of the patients with NMDs. For this purpose, the scores were transformed into Z-scores. The level of significance was set at a value of *p* < 0.05.

## 3. Results

### 3.1. Descriptive Analyses of Coping Strategies and HRQoL

Table 2 presents the results of the different HRQoL scores analyzed in the adult patients with NMD. The findings reflect worse physical health (physical SIP and standardized physical component) compared to psychosocial or mental health (psychosocial SIP and standardized mental component) among the participants. The results of the NMD patients in the different coping strategies measured using the COPE-60 test are presented in Table 2. Personal growth was the most frequently used strategy, whereas the least-used strategy was substance use.

### 3.2. Correlations between Coping Strategies and HRQoL

When analyzing the correlations obtained in the sample, the physical SIP score showed significant correlations with denial (Rho = 0.373, *p* = 0.003), restraint (Rho = 0.270, *p* = 0.035), and behavioral disengagement (Rho = 0.373, *p* = 0.008). Significant correlations were found between the psychosocial SIP and suppression of competing activities (Rho = 0.314, *p* = 0.014), venting (Rho = 0.320, *p* = 0.012), denial (Rho = 0.283, *p* = 0.027), personal growth (Rho = −0.325, *p* = 0.011), behavioral disengagement (Rho = 0.456, *p* < 0.001), and mental disengagement (Rho = 0.457, *p* < 0.001) strategies. The Total SIP showed significant correlations with suppression of competing activities (Rho = 0.257, *p* = 0.045), venting (Rho = 0.318, *p* = 0.012), denial (Rho = 0.351, *p* = 0.006), restraint (Rho = 0.264, *p* = 0.040), behavioral disengagement (Rho = 0.496, *p* < 0.001), and mental disengagement (Rho = 0.416, *p* < 0.001). Similarly, the Standardized Physical Component score of SF-36 correlated significantly with the following coping strategies: suppression of competing activities (Rho = −0.280, *p* = 0.029), venting (Rho = −0.314, *p* = 0.014), denial (Rho = −0.382, *p* = 0.002), restraint (Rho = −0.308, *p* = 0.016), behavioral disengagement (Rho = −0.453, *p* < 0.001), and mental disengagement (Rho = −0.261, *p* = 0.042). The Standardized Mental Component score presented significant correlations with seeking social support (Rho = 0.256, *p* = 0.039), suppression of competing activities (Rho = −0.282, *p* = 0.028), venting (Rho = −0.303, *p* = 0.018), behavioral disengagement (Rho = −0.352, *p* < 0.005), and mental disengagement (Rho = −0.328, *p* = 0.010). 

### 3.3. Multiple Regression Analyses between Coping Strategies and HRQoL

Multiple regression analyses were used to assess the influence of different coping strategies and degrees of disability on the HRQoL reported by the patients with NMDs (Table 3). The following variables were considered to be predictors of the model: degree of disability, seeking social support, religion, humor, venting, restraint, and mental disengagement. This model did not provide significant results for any of the HRQoL indicators analyzed: substance use, planning, suppression of competing activities, acceptance, denial, active coping, and personal growth.

## 4. Discussion

This study examined the use of different coping strategies in a sample of adults with NMDs. In addition, the influences of different coping strategies and degrees of disability on their HRQoL were also examined. 

According to the results, the most commonly used coping strategies were personal growth, acceptance, and planning. In contrast, denial, religion, and substance use were the least used. The data obtained are very similar to those found in the study by Schlüter et al. [27] conducted on ALS patients. This study found that acceptance, personal growth, and planning, among others, were the most widely used strategies by the participants, while substance use, religion, and denial were the least prevalent. Likewise, a study conducted by Carnero Contentti et al. [44] showed that acceptance and active coping strategies were the most used by patients with multiple sclerosis. Considering the findings obtained in this study, it should be highlighted that a combination of both types of coping strategies, both problem-centered and emotion-centered, is considered an adaptive way of coping with a stressful situation [45]. However, it could not be clarified whether emotion-centered strategies were more predominant compared to problem-centered ones in patients with different types of NMDs [9,24,30]. While both of these groups of strategies are legitimate, emotion-centered strategies tend to appear most often when, after assessing the situation, individuals decide that they cannot do anything to change what is harmful or threatening to them. Instead, individuals tend to adopt problem-focused coping strategies when they perceive that their efforts to try to change the situation are likely to be effective [22]. 

With regards to HRQoL, the results indicated that not only the physical dimension but also the psychosocial or mental dimension were affected, with the physical one being the most reduced. This is in line with previous studies reporting that the physical health dimension of HRQoL was the most affected in patients diagnosed with different NMDs [46,47,48,49]. Nevertheless, some authors have recently suggested that impairments in mental function are the most significant predictors of HRQoL [33].

The regression models suggested that the degree of disability was a relevant variable with impacts on HRQoL, since it appeared as part of the model that allowed us to explain the variance of these variables in both the physical and psychosocial HRQoL scores. Therefore, it is understood that the degree of disability has an important effect on the patients’ HRQoL, resulting in a greater degree of disability, with negative consequences in the HRQoL. Similar results were found in other studies [25,33] which demonstrated that an increase in the degree of disability in patients with muscular dystrophies produced a significant deterioration in HRQoL.

However, for the mental HRQoL score, the degree of disability did not appear as part of the explanatory model and was therefore not considered to be a determining variable in the worsening of this HRQoL dimension. This initially suggests that, apart from the physical component, other different variables can influence different HRQoL dimensions. 

Regarding coping strategies, it was found that restraint, mental disengagement, venting, humor, and religion all had negative influences on HRQoL. In fact, restraint was the only strategy that explained the physical domain of HRQoL. Restraint coping involves making sure that one does not respond to stress in a reactive way [50]. This relationship has been found in another study in which the use of restraint coping was related to poorer physical functioning [51]. In this regard, following the classification of adaptive vs. non-adaptive coping strategies made by Meyer [52], it is confirmed that non-adaptive strategies resulted in the worst HRQoL in its different dimensions (restraint, mental disengagement, venting, and humor). The use of non-adaptive strategies as a predictor of a worse HRQoL was also reported by Carnero Contentti et al. [44]. However, this could not be said of the religion strategy, as it is considered to be an adaptive strategy [52]. The results obtained are somewhat contradictory to the evidence reported by van Groenestijn et al. [32], for whom religiosity was associated with better HRQoL levels. On the other hand, the seeking social support strategy was the only one that maintained a positive causal relationship with HRQoL. More specifically, seeking social support is a form of coping that improves HRQoL in psychosocial terms, although this could not be observed in the other physical or psychological dimensions of HRQoL. Therefore, finding what form of social support seeking predicts a better HRQoL in this sample is in line with what Lazarus and Folkman [22] described about this coping strategy. Social support refers to emotional elements and instrumental support from members of the social network, such as family, friends, and health professionals. This coping strategy can affect how individuals adapt behaviorally and psychologically to chronic illness [53]. It is a crucial factor in coping with illness and a variable that can reduce the impact of the disease [54]. The fact that they are chronic means that the patients will need support in performing certain activities of daily living and, therefore, seeking social support will improve their performance of these activities. In addition, seeking social support can lead to a reduction in negative feelings due to the possibility of sharing their experiences with other people and emotionally relying on them [55]. Finally, social support has been shown to be a specifically relevant aspect of the lives of NMD patients, which allows us to have a better understanding of their social functioning and health experience [56].

Although this study may shed light on the use of different coping strategies and the impact this has on the HRQoL of patients diagnosed with different NMDs, various limitations of this study may be noted. Firstly, the small size of the sample is noteworthy, which limits the capacity of representativeness of the results and their application to the general clinical population. In addition, a sample size calculation was not conducted due to the lack of data available for rare NMDs. The design of this study also involves a limitation, since the recruitment of the sample was performed for convenience and a control group of people without NMD was not included. Likewise, it should be noted that a large part of the sample was made up of patients with MG, which affects the homogeneity of the sample. Furthermore, other clinical psychological variables that may have a relevant interaction with the coping style, such as depression or anxiety, were considered [19,44]. Finally, as a proposal for future research, it could be interesting to analyze differences in coping styles according to age and how this would reflect potential impacts on HRQoL. Authors like Hugel et al. [57] found that those participants classified as “non-copers” were younger and had significantly higher levels of anxiety and depression. Along these lines, it would be interesting to compare the coping styles used by different patients according to the time elapsed since the onset of their symptoms and the signs of the disease appearing.

## 5. Conclusions

To conclude, this study shows that people affected by NMDs tend to use coping strategies such as personal growth, planning, and acceptance. Likewise, a higher level of disability and coping strategies such as restraint, mental disengagement, venting, humor, and religion negatively affected HRQoL. Social support can be considered to be the main coping strategy that leads to an improvement in HRQoL. Hence, the main contributions of this study are both the identification of the most widely used coping strategies and their impact on HRQoL. These findings are relevant for clinical practice, given the need to understand patients’ coping variables to improve their HRQoL.

## Figures and Tables

**Table 1:** Sociodemographic and clinical data of the sample.

		NMD Patients (*n* = 61)	
		Mean (95% CI)	
		*n* (%)	*SD*
**Age (yrs)**		49.33 (46.20–52.52)	12.33
**Women/men**		38 (62.3%)/23 (37.7%)	
**Type of NMD**			
	MG	39 (63.9%)	
	FSH	6 (9.8%)	
	BMD	3 (4.9%)	
	LGMD	7 (11.5%)	
	HSP	1 (1.6%)	
	EDMD	1 (1.6%)	
	CMT	1 (1.6%)	
	Dermatomyositis	1 (1.6%)	
	SMA	2 (3.3%)	
**Degree of disability reported**			
	Mild	19 (31.1%)	
	Moderate	25 (41%)	
	Severe	10 (16.4%)	
	No problem	7 (11.5%)	

*Note.* CI = confidence interval; *n* = number of participants; % = percentage of participants; *SD* = standard deviation; MG = myasthenia gravis; FSH = facioscapulohumeral dystrophy; BMD = Becker muscular dystrophy; LGMD = limb–girdle muscular dystrophy; HSP = hereditary spastic paraplegia; EDMD = Emery–Dreifuss dystrophy; CMT = Charcot–Marie–Tooth disease; SMA = spinal muscular atrophy.

**Table 2:** Descriptive analysis of quality of life and coping strategies.

			NMD Patients (*n* = 61)	
			Mean (95% CI)	*SD*
*HRQoL*	**SIP**			
		Physical	19.92 (0.00–48.89)	15.20
		Psychosocial	17.65 (0.00–54.17	15.71
		Total	19.87 (0.00–47.06	12.54
	**SF-36**			
		Standardized physical component	34.03 (13.73–54.78)	10.51
		Standardized mental component	44.99 (16.78–67.23)	13.22
*Coping strategies*	**COPE-60**			
		Personal growth	2.94 (2.76–3.13)	0.71
		Acceptance	2.82 (2.65–3.00)	0.69
		Planning	2.50 (2.33–2.67)	0.67
		Positive reinterpretation	2.49 (2.36–2.62)	0.52
		Seeking social support	2.39 (2.22–2.57)	0.68
		Venting	2.32 (2.13–2.52)	0.77
		Restraint	2.31 (2.16–2.47)	0.60
		Active coping	2.11 (1.98–2.26)	0.56
		Behavioral disengagement	2.01 (1.85–2.17)	0.63
		Humor	1.64 (1.45–1.82)	0.72
		Mental disengagement	1.53 (1.40–1.67)	0.51
		Suppression of competing activities	1.48 (1.32–1.64)	0.62
		Denial	1.45 (1.34–1.57)	0.46
		Religion	1.42 (1.25–1.59)	0.66
		Substance use	1.08 (1.00–1.16)	0.30

*Note.* CI = confidence interval; *n* = number of participants; % = percentage of participants; *SD* = standard deviation; HRQoL = health-related quality of life; SIP = The Sickness Impact Profile test; SF-36 = The 36-item Short form Health Survey test; COPE-60 = Coping Orientations to Problems Experienced test.

**Table 3:** Multiple linear regressions of the predictive and potentially HRQoL-related variables in the sample of patients with NMDs.

		Physical SIP	Psychosocial SIP	Total SIP	Standardized Physical Component	Standardized Mental Component
R^2^		0.591	0.416	0.600	0.512	0.355
Adj. R^2^		0.577	0.396	0.586	0.495	0.309
Standardized regression coefficients (β)						
Degree of disability		0.730 **	0.396 **	0.641 **	−0.660 **	-
Seeking social support		-	-	-	-	0.503 **
Religion		-	-	-	-	−0.229 *
Humor		-	-	-	-	−0.345 *
Substance use		-	-	-	-	-
Planning		-	-	-	-	-
Suppression of competing activities		-	-	-	-	-
Venting		-	-	-	-	−0.430 **
Acceptance		-	-	-	-	-
Denial		-	-	-	-	-
Restraint		0.192 *	-	-	−0.232 *	-
Active coping		-	-	-	-	-
Personal growth		-	-	-	-	-
Mental disengagement		-	0.400 **	0.278 *	-	-

*Note.* * *p* < 0.05; ** *p* < 0.001.

## Data Availability

The datasets generated and/or analyzed during the current study are not publicly available because they belong to the University of Deusto, but they are available from the corresponding author (Irune García) upon reasonable request.
